# Bilirubin Nanoparticles Reduce Diet-Induced Hepatic Steatosis, Improve Fat Utilization, and Increase Plasma β-Hydroxybutyrate

**DOI:** 10.3389/fphar.2020.594574

**Published:** 2020-12-18

**Authors:** Terry D. Hinds, Justin F. Creeden, Darren M. Gordon, Donald F. Stec, Matthew C. Donald, David E. Stec

**Affiliations:** ^1^ Department of Pharmacology and Nutritional Sciences, University of Kentucky College of Medicine, Lexington, KY, United States; ^2^ Department of Neurosciences, University of Toledo College of Medicine, Toledo, OH, United States; ^3^ Small Molecule NMR Facility Core, Vanderbilt Institute of Chemical Biology, Vanderbilt University, Nashville, TN, United States; ^4^ Department of Physiology and Biophysics, Cardiorenal and Metabolic Diseases Research Center, University of Mississippi Medical Center, Jackson, MS, United States

**Keywords:** obesity, non-alcoholic fatty liver disease, ketone, ketosis, apolipoprotein, heme oxygenase, HO-1

## Abstract

The inverse relationship of plasma bilirubin levels with liver fat accumulation has prompted the possibility of bilirubin as a therapeutic for non-alcoholic fatty liver disease. Here, we used diet-induced obese mice with non-alcoholic fatty liver disease treated with pegylated bilirubin (bilirubin nanoparticles) or vehicle control to determine the impact on hepatic lipid accumulation. The bilirubin nanoparticles significantly reduced hepatic fat, triglyceride accumulation, *de novo* lipogenesis, and serum levels of liver dysfunction marker aspartate transaminase and ApoB100 containing very-low-density lipoprotein. The bilirubin nanoparticles improved liver function and activated the hepatic *β*-oxidation pathway by increasing PPARα and acyl-coenzyme A oxidase 1. The bilirubin nanoparticles also significantly elevated plasma levels of the ketone *β*-hydroxybutyrate and lowered liver fat accumulation. This study demonstrates that bilirubin nanoparticles induce hepatic fat utilization, raise plasma ketones, and reduce hepatic steatosis, opening new therapeutic avenues for NAFLD.

## Introduction

Obesity is at an all-time high, and this is prevalent worldwide. The tissue overload from lipids in the obese may cause other comorbidities such as non-alcoholic fatty liver disease (NAFLD), insulin-resistant diabetes, cardiovascular disease, and some cancers (John et al., 2016; Lega and Lipscombe, 2020). Therapeutic inventions for reducing obesity are limited, and most increase blood pressure and may further complicate the cardiovascular outcomes. We and others have previously shown that the heme metabolite, bilirubin, reduce fat accumulation and blood glucose levels in obese mice (Dong et al., 2014; Hinds et al., 2014; Liu et al., 2015; Gordon et al., 2016; Hinds et al., 2016; Stec et al., 2016; Hinds et al., 2017; Takei et al., 2019; Stec et al., 2020). Bilirubin offers a promising therapeutic approach as it benefits the cardiovascular system (Hinds and Stec, 2018; Hinds and Stec, 2019) by preventing hypertension (Vera et al., 2009) and improving blood flow (Vera and Stec, 2010). Bilirubin’s actions to reduce lipid accumulation have been attributed to the recent findings that it has a hormonal function by binding directly to the nuclear receptor peroxisome proliferator-activated receptor *α* (PPARα), which induces gene transcription that promotes fat burning (Gordon et al., 2016; Stec et al., 2016; Gordon et al., 2019).

Not surprisingly, mice with a hepatocyte-specific deletion of PPARα develop hepatic steatosis and NAFLD that is worsened on a high-fat diet (Stec et al., 2019). PPARα induces genes for *β*-oxidation and fat utilization, which reduces hepatic fat and NAFLD (Burri et al., 2010; Grabacka et al., 2016). During this process, PPARα regulates processes that mediate ketone body production from fatty acid oxidation that increases serum levels of the ketone *β*-hydroxybutyrate (BOHB) (Kersten et al., 1999; Grabacka et al., 2016). The BOHB is excreted by the liver to the blood and serves as a fuel source for the body other than glucose (Newman and Verdin, 2014a; Newman and Verdin, 2014b). Fat accumulation in the liver suppresses *β*-oxidation and reduces ketone production lowering plasma BOHB. Mey et al. found that BOHB levels are reduced in humans with obesity-related NAFLD (Mey et al., 2020). While it was not a major conclusion in the paper, they also found that bilirubin levels were lower in patients with NAFLD. Others have shown that plasma bilirubin levels are negatively associated with NAFLD (Hjelkrem et al., 2012; Puri et al., 2013; Salomone et al., 2013). While patients exhibiting mildly elevated bilirubin levels had significantly less NAFLD indicating an inverse relationship (Kwak et al., 2012). These studies suggest that bilirubin and BOHB may be positively correlated; however, no studies have shown that when bilirubin is elevated that BOHB is also higher.

While large-scale population studies have associated the protective effects of plasma bilirubin levels on NAFLD development, translating these findings into therapies to patients has been complicated. One of the main reasons for this difficulty is the lack of formulations of bilirubin that could be administered to human patient populations. Bilirubin is a very hydrophobic molecule and does not dissolve in aqueous solutions easily. This property limits its use in patients because solvents are customarily required to get bilirubin into solutions such as saline and others used. One resolution to this problem is to covalently attach a polyethylene glycol compound to bilirubin to form pegylated bilirubin (PEG-BR) and promote the formation of highly water-soluble bilirubin nanoparticles (Zheng et al., 2019). PEG-BR has been an effective anti-inflammatory and anti-oxidant in several *in vivo* models (Lee et al., 2016; Kim M. J. et al., 2017). However, its effectiveness as a potential therapeutic for NAFLD has not been evaluated.

Here, we wanted to determine bilirubin’s functionality on obesity-induced hepatic steatosis and NAFLD and determine whether it activates *β*-oxidation, fat utilization, and BOHB production using bilirubin nanoparticles. We found that hepatic PPARα induced and liver fat content was lower, which correlated with higher plasma BOHB levels and lower serum triglycerides. Our results demonstrate a possible role for bilirubin nanoparticles in the protection against obesity-induced fatty liver disease.

## Materials and Methods

Animals. The experimental procedures and protocols of this study conformed to the National Institutes of Health Guide for the Care and Use of Laboratory Animals and approved by the Institutional Animal Care and Use Committee of the University of Mississippi Medical Center. C57BL/6J mice were purchased from Jackson Labs (Bar Harbor, ME, United States) and placed on a 60% high-fat diet (diet #D12492, Research Diets, Inc., New Brunswick, NJ, United States) for 24 weeks with full access to tap water. After this time, mice were randomly assigned to either a treatment group consisting of pegylated bilirubin nanoparticles (30 mg/kg every other day, i.p.) or vehicle (saline) for 4 weeks while continuing on the high-fat diet.

Pegylated bilirubin synthesis. The synthesis of PEG-BR was done at the Research Institute of Pharmaceutical Sciences at the University of Mississippi (Oxford, MS, United States). The PEG-BR was prepared from bilirubin-IX-alpha (Frontier Scientific, Logan, UT, United States) and mPEG2000-NH2 (Sigma-Aldrich, St. Louis, MO, United States) as previously described (Gordon et al., 2016; Lee et al., 2016; Kim M. J. et al., 2017). The size and morphology of PEG-BR were analyzed by transmission electron microscopy (TEM) using a model JEM-2100 (JEOL Ltd., Tokyo, Japan). Purity of the PEG-BR was found to be 95%. PEG-BR was resuspended in saline with slight sonication to dissolve and stored at −20°C in the dark.

Liver composition. Liver composition was measured at the end of the study using magnetic resonance imaging (EchoMRI-900TM, Echo Medical System, Houston, TX, United States). MRI measurements were performed on whole livers placed in a thin-walled plastic cylinder. Liver fat and lean mass were obtained and expressed as a percent of total liver weight.

Liver triglyceride measurement. Triglycrides were measured from 100 mg of liver tissue homogenized in 1 ml of 5% NP-40 in water. Homogenized tissues were then heated to 95°C for 5 min and then centrifuged (13,000 × *g*) for 2 min. Tissue triglyceride levels were measured using a colorimetric assay kit according to manufactures’ guidelines (Triglyceride Quantification Colorimetric/Fluorometric Kit, BioVision, Milpitas, CA, United States). Tissue triglyceride are expressed as mM. Samples from individual mice were run in duplicate and averaged, and the averages used to obtain group averages.

Liver histology. To determine hepatic differences of the pegylated bilirubin and vehicle treated mice, livers were mounted and frozen in Tissue-Tek O.C.T and sectioned at 10 µm. Hematoxylin and Eosin (H&E) staining were performed as previously described (Hinds et al., 2016; Hinds et al., 2017; Stec et al., 2019). The Oil Red O (CAS Number 1320-06-5, Sigma-Aldrich, St. Louis, MI, United States) staining was performed on 10 µm thick formalin-fixed livers. The livers were stained with freshly prepared Oil Red O working solution 15 min, rinsed with 60% isopropanol, and nuclei stained with alum hematoxylin. Then, slides were rinsed with distilled water and mounted in aqueous mountant and prepared for imaging. The degree of Oil Red O staining was determined at 20× magnification using a color video camera attached to an Olympus VS120 slide scanning microscope (Olympus Corporation, Center Valley, PA, United States). Images were analyzed using the Olympus OlyVIA software. Image J (NIH) was used to quantitate the lipid droplets. Data are presented as the ±SEM of the Oil Red O staining for each group.

AST/ALT measurements. Plasma alanine transaminase (ALT) and aspartate transaminase (AST) were measured using a Vet Axcel serum chemistry analyzer (AlfaWassermann, West Caldwell, NJ, United States) from 30 μL of plasma. Samples were measured in duplicate with standards supplied by the manufacturer. Data are presented as Units (U)/L.

Quantitative Real-Time PCR Analysis. Total RNA was harvested from the animals by lysing livers using a Qiagen Tissue Lyser LT (Qiagen Inc., Germantown, MD, United States) and then extraction by 5-Prime PerfectPure RNA Tissue Kit (Thermo Fisher Scientific, Wilmington, DE, United States). Total RNA was read on a NanoDrop 2,000 spectrophotometer (Thermo Fisher Scientific, Wilmington, DE, United States) and cDNA was synthesized using High Capacity cDNA Reverse Transcription Kit (Applied Biosystems, Foster City, CA, United States). PCR amplification of the cDNA was performed by quantitative real-time PCR using TrueAmp SYBR Green qPCR SuperMix (Alkali Scientific, Fort Lauderdale, FL, United States) for gene-specific primers as previously described (Hinds et al., 2011; Hinds et al., 2014; Hinds et al., 2016; Marino et al., 2016; Stec et al., 2016; Hinds et al., 2017). The thermocycling protocol consisted of 5 min at 95°C, 40 cycles of 15 s at 95°C, and 30 s at 60°C, finished with a melting curve ranging from 60 to 95°C to allow distinction of specific products. Normalization was performed in separate reactions with primers to GAPDH.

Gel Electrophoresis and Western Blotting—Mouse tissues were flash frozen in liquid nitrogen during harvesting and stored at −80°C. For gel electrophoresis, 50–100 mg of cut tissue was then resuspended in three volumes of CelLytic Buffer (Sigma-Aldrich, St. Louis, MO, United States, Cat No. C3228) plus 10% protease inhibitor cocktail (Sigma-Aldrich, St. Louis, MO, United States, Cat No. P2714-1BTL) and Halt phosphatase inhibitor cocktail (Thermo Fisher Scientific, Wilmington, DE, United States, Cat No. PI78420), and then incubated on ice for 30 min. The livers were lyzed using a Qiagen Tissue Lyser LT (Qiagen Inc., Germantown, MD, United States) and then centrifuged at 100,000 × *g* at 4°C. Protein samples were resolved by SDS polyacrylamide gel electrophoresis and electrophoretically transferred to Immobilon-FL membranes. Membranes were blocked at room temperature for 2 h in TBS (10 mM Tris-HCl (pH 7.4) and 150 mM NaCl) containing 3% BSA. Subsequently, the membranes were incubated overnight at 4°C with the following antibodies: ACOX1 (Santa Cruz Biotechnology, Santa Cruz, CA, sc-98499), fatty acid synthase (FAS) (Cell Signaling Technology, Danvers, MA, United States, Cat No. 3180S), SCD1(Cell Signaling Technology, Danvers, MA, United States, Cat No. 2794S), or heat shock protein 90 (HSP90) (Santa Cruz, sc-13119). After three washes in TBS + 0.1% Tween 20, the membrane was incubated with an infrared anti-rabbit (IRDye 800, green) or anti-mouse (IRDye 680, red) secondary antibody labeled with IRDye infrared dye (LI-COR Biosciences) (1:10,000 dilution in TBS) for 2 h at 4°C. Immunoreactivity was visualized and quantified by infrared scanning in the Odyssey system (LI-COR Biosciences).

Analysis of plasma lipids and metabolites. Plasma lipids and metabolites were measured in mice following an 8 h fast by nuclear magnetic resonance (NMR) spectroscopy as part of the Bruker IVDr platform (Bruker Scientific LLC, Billerica, MA, United States), as previously described (Stec et al., 2019). Plasma samples (50 μL) were combined with 150 μL of buffer supplied by Bruker Biospin specifically for the IVDr protocol and were analyzed according to the Bruker *In-Vitro* Diagnostics research (IVDr) protocol. Lipoprotein subclass analysis was performed using regression analysis of the NMR data as previously described (Stec et al., 2019).

Statistics. All bar graph data are presented as mean ± S.E.M. Box and whisker plots display whiskers from the minimum or maximum, with a vertical line in the box to indicate the median. Differences between treatment groups were determined using student t-test or one-way analysis of variance with a post hoc test (Dunnett’s). A *p* < 0.05 was considered to be significant. All analyses were performed with GraphPad Prism eight software (GraphPad Software, Inc., San Diego, CA).

## Results

**FIGURE 1:**
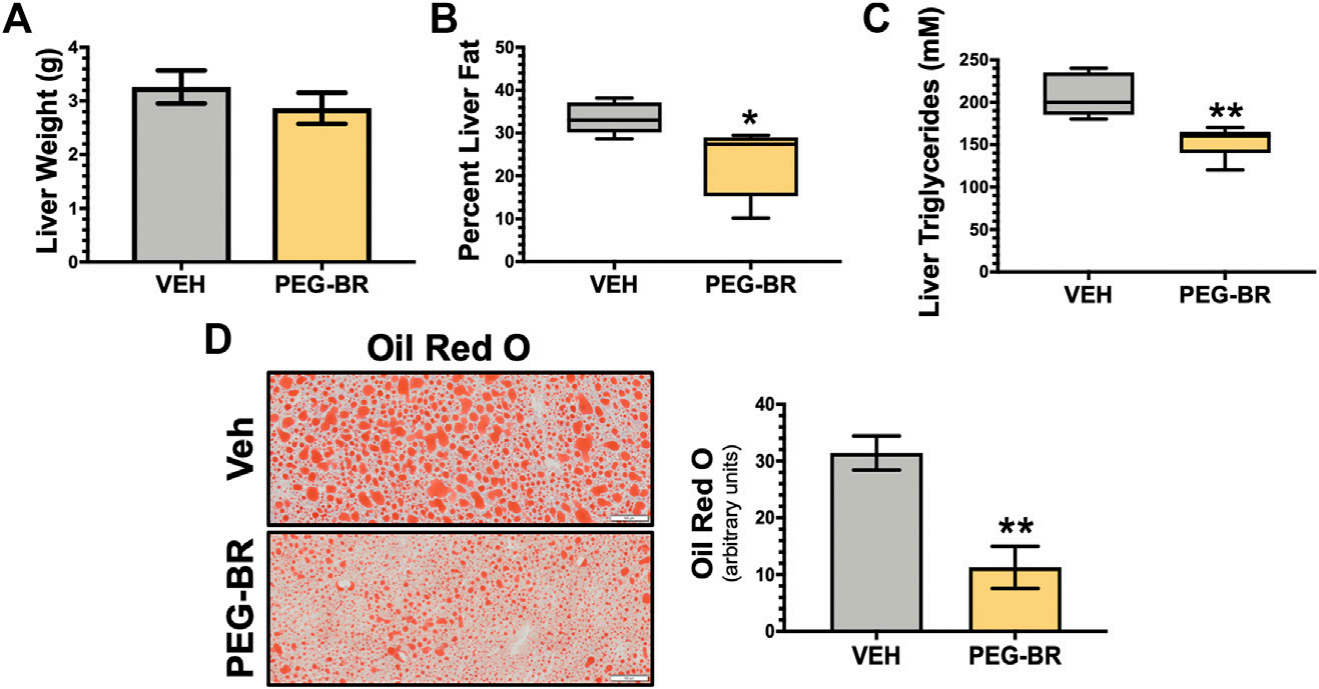
Bilirubin nanoparticles reduce hepatic fat content. Liver weight (*g*) **(A)**, percent liver fat as determined by EchoMRI **(B)**, hepatic triglycerides (mM) **(C)**, and hepatic Oil Red O staining **(D)** in vehicle (VEH) and pegylated bilirubin (PEG-BR) treated mice. White scale bar = 100 μm. * = *p* < 0.05 vs. VEH; ** = *p* < 0.01 vs. VEH; (VEH, *n* = 5 and PEG-BR, *n* = 6).

Bilirubin has almost exclusively been correlated with liver dysfunction. The recent findings that bilirubin is inversely associated with NAFLD (Jang, 2012; Kwak et al., 2012) and that a mouse model of Gilbert’s syndrome with hyperbilirubinemia were resistant to hepatic steatosis (Hinds et al., 2017), has provided new insights into its function. We wanted to determine if bilirubin nanoparticles (pegylated bilirubin) that we and others have recently described (Gordon et al., 2016; Lee et al., 2016; Kim et al., 2017a; Kim M. J. et al., 2017; Lee et al., 2019) could improve hepatic steatosis in an obese mouse model. As previously described (Gordon et al., 2016), we put mice on a high-fat diet (HFD) for 24 weeks and then treated for 4-weeks with PEG-BR or vehicle while maintaining them on a HFD. The obese mice’s body weights at the 24-weeks point were comparable, with the starting weight of the mice over 50 g for each group (55.7 ± 4.1 g Vehicle vs. 53.4 ± 2.6 PEG-BR treated) (*p* = 0.2823). After the 4-weeks treatment, the plasma bilirubin levels were increased in the PEG-BR treated (0.45 ± 0.08 mg/dl) compared to the vehicle (0.13 ± 0.05 mg/dl) (*p* < 0.0001). After the 4-weeks PEG-BR and vehicle treatments, the percent body weight change was greater in the vehicle but not the PEG-BR (103.4% ± 2.5 for vehicle and 96.3% ± 2.7 for PEG-BR) (*p* = 0.0058). The PEG-BR group had a 7% reduction in body weight gain compared to the vehicle treated animals. There was no difference between the groups for the liver weight (Figure 1A). The liver to body weight ratio was 0.056 ± 0.004 vs. 0.055 ± 0.004 g/g Vehicle vs. PEG-BR treated. However, percent liver fat measured by echoMRI, liver triglycerides, and lipid Oil Red O staining all showed less fat accumulation in the PEG-BR treated animals compared to vehicle (Figures 1B–D).

**FIGURE 2:**
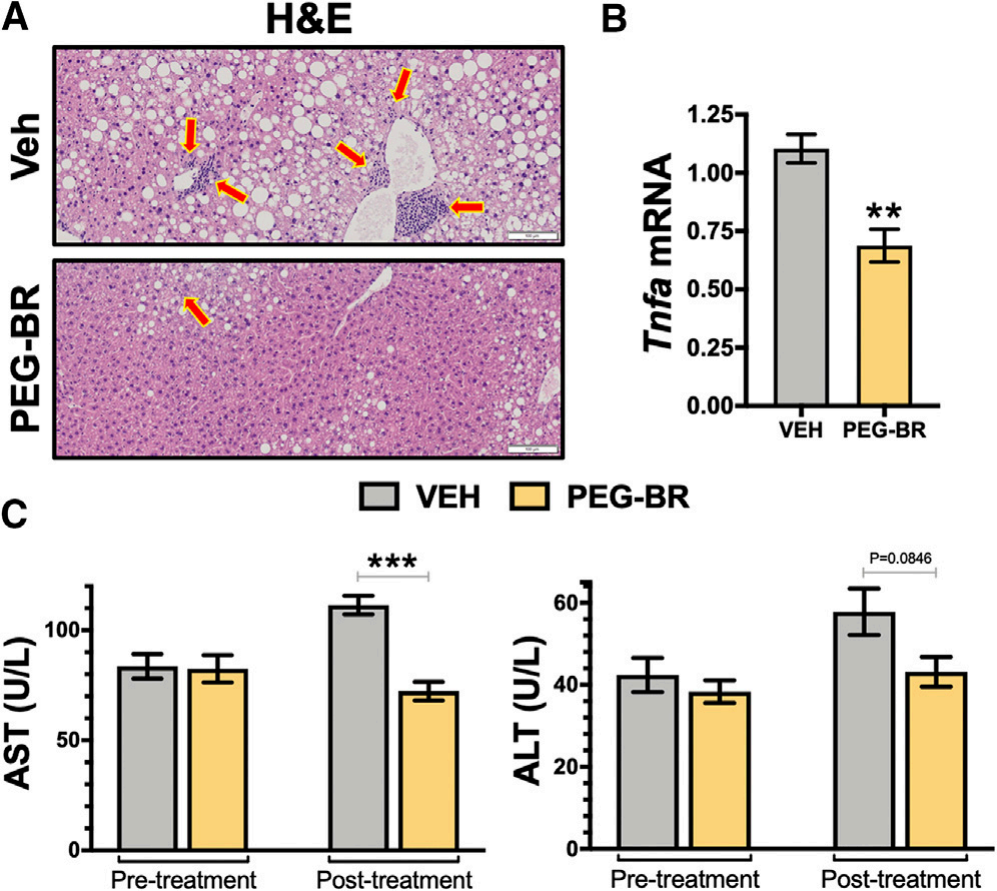
Bilirubin nanoparticles improve hepatic function and inflammation in obese mice. Hematoxylin and eosin (H&E) staining in vehicle (VEH) and pegylated bilirubin (PEG-BR) treated mice **(A)**. Real-time PCR expression of hepatic tumor necrosis factor alpha (*Tnfa*) **(B)**; ** = *p* < 0.001 vs. VEH; (VEH, *n* = 4 and PEG-BR, *n* = 4). Liver dysfunction markers alanine transaminase (ALT) and aspartate transaminase (AST) plasma levels were measured at the beginning (pre-treatment) of the study and after the 4-weeks treatment was completed (post-treatment); ** = *p* < 0.01 vs. VEH; (VEH, *n* = 5 and PEG-BR, *n* = 6).

To better understand how the bilirubin nanoparticles impact hepatic function, the livers from both groups of mice were analyzed by hematoxylin and eosin (H&E) staining for observable differences in treated animals and hepatic dysfunction biomarkers. The H&E staining revealed that the VEH treated animals possibly had inflammation, which were found to be lower in the PEG-BR treated group (Figure 2A). This was paralleled with pro-inflammatory gene *Tnfa*, in that PEG-BR significantly (*p* < 0.01) reduced expression compared to VEH (Figure 2B). We measured plasma levels of hepatic dysfunction markers alanine transaminase (ALT) and aspartate transaminase (AST) before (pre-treatment) treatment and after the study was completed (post-treatment). There were no differences in AST or ALT levels at the beginning of the study (Figure 2C). However, post-treatment, the AST levels were significantly (*p* < 0.001) reduced with PEG-BR compared to VEH groups. The ALT level was also reduced with PEG-BR was not reduced but showed a trend toward reduction that did not reach statistical significance. Overall, these data demonstrate that PEG-BR improved hepatic function and reduced inflammation.

**FIGURE 3:**
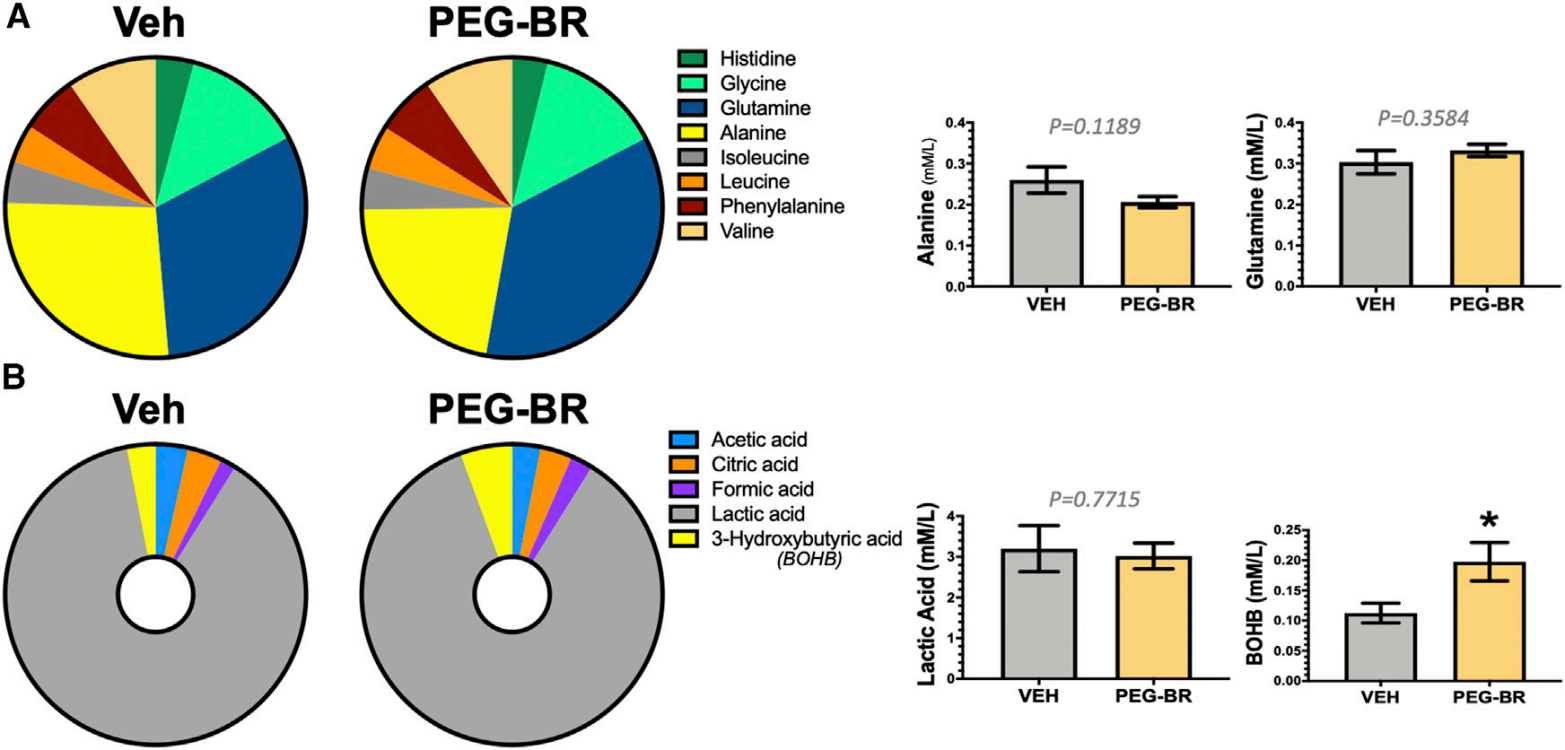
Plasma metabolites in bilirubin nanoparticles and vehicle treated obese mice. Pie chart and bar graphs of plasma amino acids in vehicle and pegylated bilirubin treated mice **(A)**. Pie chart and bar graphs of plasma carboxylic and keto acids in vehicle and pegylated bilirubin treated mice **(B)**. * = *p* < 0.05 vs. VEH; (VEH, *n* = 3 and PEG-BR, *n* = 4–5).

**FIGURE 4:**
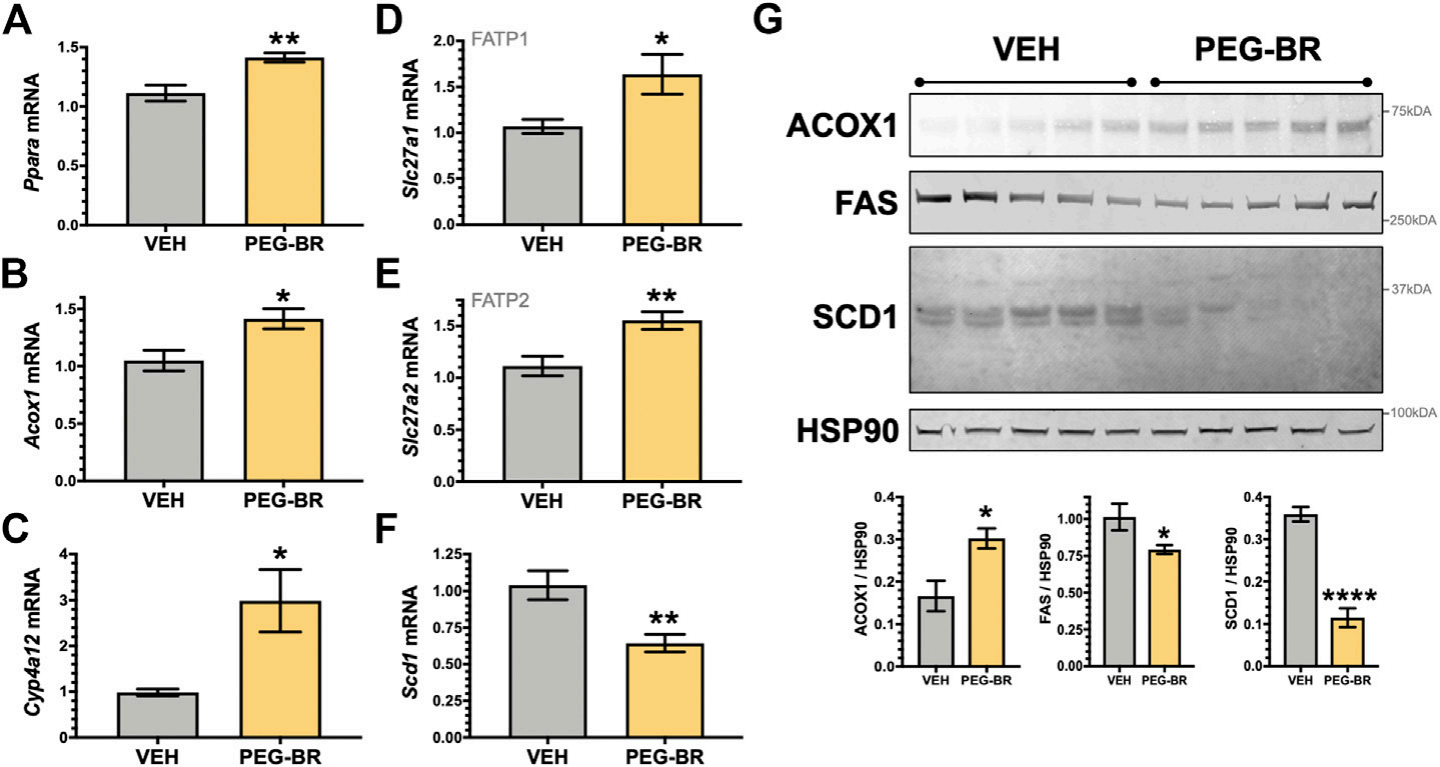
Hepatic PPARα and target gene expression in bilirubin nanoparticle and vehicle treated obese mice. Real-time PCR expression of hepatic PPARα (*Ppara*) **(A)** and target genes, acyl-Coenzyme A oxidase 1 (*Acox1*) **(B)**; cytochrome P450, family 4, subfamily a, polypeptide 12a (*Cyp4a12*) **(C)**; FATP1, solute carrier family 27 member 1 (*Slc27a1*) **(D)**; and, FATP2, solute carrier family 27 member 2 (*Slc27a2*) **(E)**; stearoyl-Coenzyme A desaturase 1 (*Scd1*) **(F)** in vehicle and pegylated bilirubin treated mice. * = *p* < 0.05 vs. VEH; ** = *p* < 0.001 vs. VEH. (VEH, *n* = 5 and PEG-BR, *n* = 6). **(G)** Immunoblotting of acyl-Coenzyme A oxidase 1 (ACOX1), fatty acid synthase (FAS), stearoyl-Coenzyme A desaturase 1 (SCD1), and heat shock protein 90 (HSP90) in vehicle and pegylated bilirubin treated mice. * = *p* < 0.05; ****=<0.0001 vs. VEH; (VEH, *n* = 5 and PEG-BR, *n* = 5).

Next, we determined how PEG-BR treatments affect plasma metabolites of amino acids by NMR spectroscopy using the Bruker IVDr platform. We found no significant differences in amino acids measured in the plasma of the PEG-BR or vehicle-treated groups (Figure 3A). Exercise and fat burning metabolites lactic acid, acetic acid, formic acid, and *β*-hydroxybutyrate (3-hydroxybutyric acid) were measured in the two groups. Analysis showed that only elevated plasma BOHB levels had a significant (*p* < 0.05) difference for PEG-BR treated compared to the vehicle groups (Figure 3B). There were no differences between the lactic acid, formic acid, citric acid, and acetic acid between the groups. The PEG-BR reduces hepatic lipids possibly by inducing *β*-oxidation, which provide metabolites for ketone production. Therefore, we measured known mediators that regulate fatty acid *β*-oxidation, such as PPARα and target genes. PPARα (*Ppara*) mRNA expression was increased in the PEG-BR treated animals compared to the vehicle group (Figure 4A). Huang e*t al.* showed that sustained activation of PPARα by endogenous ligands in obese mice increases the rate-limiting mediator of *β*-oxidation, *Acox1* (Vluggens et al., 2010), and p450 *Cyp4a* pathways (Hirai et al., 2007; Huang et al., 2012). Here, we also found that *Acox1* and cytochrome P450 *Cyp4a12* (Figures 4B,C) were significantly (*p* < 0.05) higher in the PEG-BR compared to vehicle-treated animals. Also, the PPARα-target genes for long-chain fatty acid transporters FATP1 (gene *Slc27a1*) and FATP2 (gene *Slc27a2*) (Hirai et al., 2007), were significantly increased in the PEG-BR treated mice compared to vehicle (Figures 4D,E). The lipogenesis gene, *Scd1*, was significantly (*p* < 0.01) reduced with PEG-BR (Figure 4F). Immunoblotting of ACOX1 showed that the protein was significantly (*p* = 0.0137) higher in the PEG-BR treated animals compared to the vehicle (Figure 4G). The *de novo* lipogenesis proteins fatty acid synthase (FAS) and stearoyl-Coenzyme A desaturase 1 (SCD1) were significantly (*p* < 0.05 and *p* < 0.0001, respectively) reduced with PEG-BR compared to vehicle treatments. These indicate that PEG-BR enhances fatty acid uptake and lipid utilization for hepatic *β*-oxidation increasing plasma BOHB and inhibits *de novo* lipogenesis, improving fatty liver.

**FIGURE 5:**
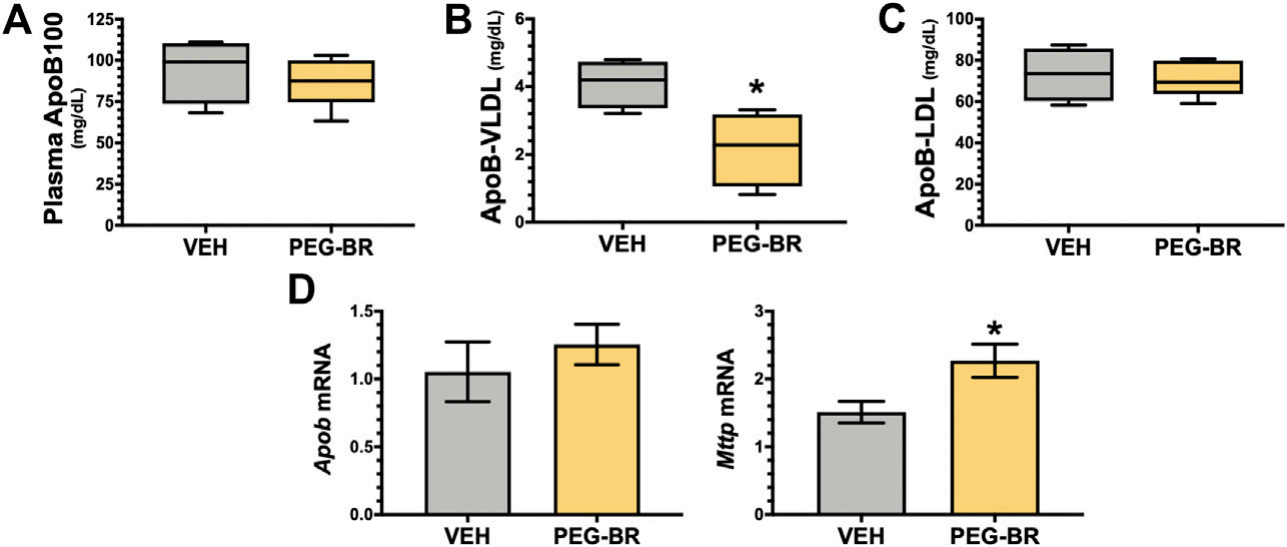
Apolipoprotein B levels in bilirubin nanoparticle and vehicle treated obese mice. Plasma apolipoprotein B levels in vehicle and pegylated bilirubin treated mice **(A)**. Plasma apolipoprotein B-Very-low-density lipoprotein (ApoB-VLDL) **(B)**. Plasma apolipoprotein B-low-density lipoprotein (ApoB-LDL) **(C)**; * = *p* < 0.05 vs. VEH; (VEH, *n* = 4 and PEG-BR, *n* = 5). **(D)** Real time PCR of hepatic apolipoprotein B (*Apob*) and Microsomal Triglyceride Transfer Protein (Mttp) mRNA in vehicle and pegylated bilirubin treated mice. * = *p* < 0.05; **=<0.01 vs. VEH; (VEH, *n* = 5 and PEG-BR, *n* = 6).

**FIGURE 6:**
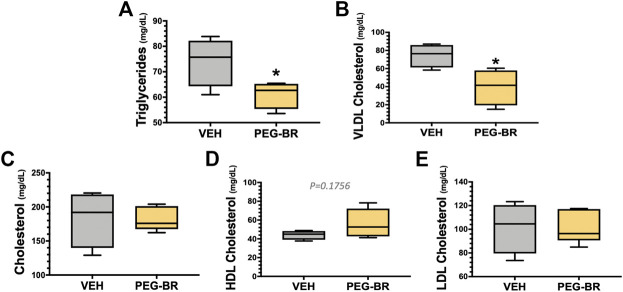
Plasma triglyceride and cholesterol levels in bilirubin nanoparticle and vehicle treated obese mice. Plasma triglycerides **(A)**, very-low-density lipoprotein (VLDL) cholesterol **(B)**, total cholesterol **(C)**, high-density lipoprotein (HDL) cholesterol **(D)**, low-density lipoprotein (LDL) cholesterol **(E)** in vehicle and pegylated bilirubin treated mice. * = *p* < 0.05 vs. VEH; (VEH, *n* = 4 and PEG-BR, *n* = 5).

**FIGURE 7:**
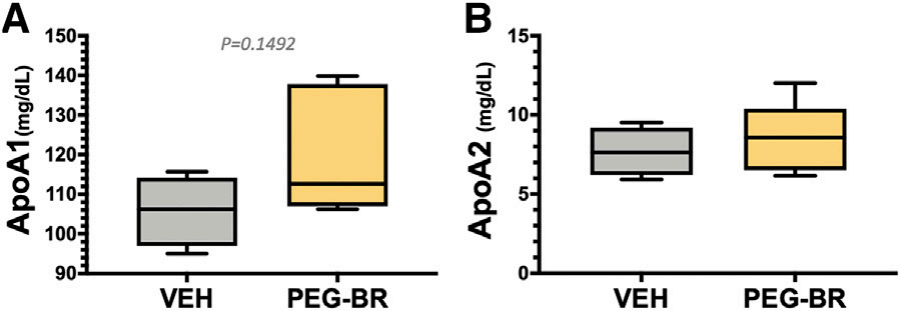
Apolipoprotein A levels in bilirubin nanoparticle and vehicle treated obese mice. Plasma apolipoprotein A1 (ApoA1) **(A)** and apolipoprotein A2 (ApoA2) **(B)** levels in vehicle and pegylated bilirubin treated mice. (VEH, *n* = 4 and PEG-BR, *n* = 5).

**FIGURE 8:**
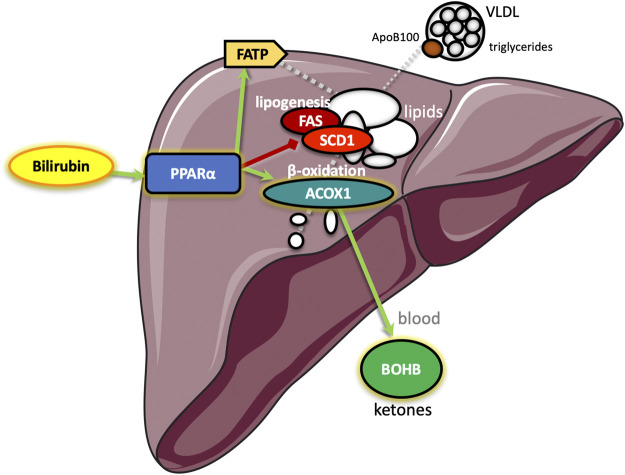
Schematic diagram of the proposed pathway by which bilirubin reduces hepatic steatosis. Bilirubin serves as a ligand to the peroxisome proliferator-activated receptor-alpha (PPARα), which increases transcription of genes for *β*-oxidation of fatty acids (acyl-Coenzyme A oxidase 1, ACOX1) resulting in metabolites that elevate hepatic production of the ketone *β*-hydroxybutyrate (BOHB), which is secreted to blood increasing levels. Bilirubin activated PPARα also stimulates hepatic fatty acid transport protein (FATP) that imports lipids to reduce blood levels and inhibits stearoyl-Coenzyme A desaturase 1 (SCD1) and fatty acid synthase (FAS) to inhibit *de novo* lipogenesis. Together, the bilirubin-PPARα controlled pathways decrease the hepatic secretion of apolipoprotein B100 (ApoB100) containing very-low-density lipoproteins (VLDL) that increase blood triglyceride levels.

We have previously shown that mice with a hepatocyte-specific deletion of PPARα (*Ppara* ^HepKO^) had worsened hepatic steatosis on a HFD that also caused significantly higher plasma triglycerides and ApoB100 levels (Stec et al., 2019). PEG-BR did not change in plasma total ApoB100 levels (Figure 5A). However, PEG-BR significantly reduced the ApoB100 containing very-low-density lipoproteins (VLDL) but not ApoB100 containing low-density lipoproteins (LDL) particles (Figures 5B,C). We had shown in the *Ppara* ^HepKO^ on HFD that had higher ApoB100 in plasma than floxed control, and that this was also correlated with reduced hepatic microsomal triglyceride transfer protein (*Mttp*) expression (Stec et al., 2019). ApoB100 and the *Mttp* are essential for excretion of the VLDL molecule from the liver (Chen et al., 2008). Here, we found that PEG-BR induced *Mttp* expression but not *ApoB* (Figure 5D). The serum ApoB-VLDL, triglycerides, and VLDL cholesterol were significantly (*p* < 0.05) lower in the PEG-BR compared to vehicle treated animals (Figures 6A,B). The triglyceride distribution panel for VLTG, IDTG, LDTG, and HDTG showed no sigificant differences, and this was also observed for the VLDL, LDL, and HDL triglyceride subfractions (Supplementary Figure S1). There was no change in total cholesterol, HDL cholesterol, or LDL cholesterol levels (Figures 6C–E). This was also observed in the cholesterol and free cholesterol distribution and HDL, LDL, and VLDL cholesterol subfractions (Supplementary Figures S2, S3). Also, lipoproteins ApoA1 and ApoA2 that remove cholesterol from peripheral tissues had no difference between the treated groups (Figures 7A,B). This was also observed in the ApoA1 and ApoA2 distribution profiles (Supplementary Figure S4). Overall, treatment of PEG-BR in obese mice improved the hepatic steatosis potentially by utilizing fat for *β*-oxidation, increasing fatty acid uptake, reducing plasma ApoB-VLDL and triglycerides (Figure 8).

## Discussion

The novel finding in our studies is that PEG-BR (bilirubin nanoparticles) improved NAFLD, which is potentially due to fat-burning mechanisms, as demonstrated by reduced hepatic triglyceride content, changes in gene expression in line with increased fat utilization and *β*-oxidation, and increased plasma levels of BHOB. Based on our current data and previous studies clearly demonstrating the induction of PPARα by bilirubin and bilirubin nanoparticles, it is likely that the effects on hepatic steatosis are at least in part mediated via PPARα regulated mechanisms. These are the first findings that PEG-BR increases plasma concentrations of BHOB. We have previously shown that bilirubin binds directly to PPARα (Gordon et al., 2016; Stec et al., 2016; Gordon et al., 2019), which is a known regulator of fatty acid *β*-oxidation (Hirai et al., 2007; Huang et al., 2012; Stec et al., 2019) that supplies the substrates for BOHB production (Kersten et al., 1999; Grabacka et al., 2016). Patients with NAFLD have lower plasma levels of BHOB (Mey et al., 2020), which is likely due to the activation of lipogenic pathways such as FAS or *Scd1* that transcribes the stearoyl-CoA desaturase-1 (SCD1) enzyme that forms fatty acids and lipid synthesis (Sampath et al., 2007; Flowers and Ntambi, 2009). A deficiency in SCD1 in mice protects against weight gain and adiposity (Ntambi et al., 2002). Activation of these pathways induces *de novo* lipogenesis for the synthesis of fat, causing the accumulation of lipids shutting down the *β*-oxidation fat burning mechanisms.

We had previously shown that mice with the Gilbert’s mutation UGT1A1*28 and hyperbilirubinemia were resistant to high-fat diet-induced hepatic steatosis by inhibiting *de novo* lipogenesis and activation of *β*-oxidation (Hinds et al., 2017). We have also shown that exercise elevated plasma bilirubin by suppressing hepatic UGT1A1 and increasing biliverdin reductase-A (BVRA) (Hinds et al., 2020), the enzyme that generates bilirubin (Adeosun et al., 2018; O’Brien et al., 2015), which improved hepatic glycogen storage and PPARα target genes (Hinds et al., 2020). Here, we found that PEG-BR reduced SCD1 and FAS while activating PPARα and ACOX1, improving hepatic steatosis in obese mice. We have also previously demonstrated that mice with a hepatocyte-specific loss of BVRA had worsened hepatic steatosis on a HFD compared to floxed control due to lower hepatic bilirubin levels (Hinds et al., 2016). We found that these mice had higher hepatic *de novo* lipogenesis and reduced *β*-oxidation via lessor bilirubin-PPARα activity. Later, a similar observation was made using murine hepatocyte cell culture with CRISPR/Cas9 deletion of BVRA (Gordon et al., 2019). Mildly elevated plasma bilirubin in obese patients may improve fatty liver and related adverse metabolic parameters such as high cholesterol or triglycerides. In line with a study by Wallner *et al.* that found humans with hyperbilirubinemia due to Gilbert syndrome have reduced serum cholesterol and triglycerides, we also showed that PEG-BR reduced VLDL cholesterol but not triglycerides (Wallner et al., 2013).

Weight gain and obesity are associated with increased plasma ApoB100 and triglyceride levels partly due to an greater in hepatic VLDL release (Chen et al., 2008; Fabbrini et al., 2016; Vine et al., 2017). PEG-BR treatment in the obese mice reduced plasma triglycerides and VLDL cholesterol, but no effect on total cholesterol levels or HDL and LDL was observed. However, the total cholesterol was slightly lower and HDL higher. The PEG-BR had a similar impact on ApoA-1 and ApoA-2 plasma levels with no changes, but a trend to increase ApoA-1 was observed. We previously showed that mice with a hepatocyte-specific deletion of PPARα (*Ppara* ^HepKO^) on a HFD had no changes in serum cholesterol, HDL, LDL, or ApoA proteins (Stec et al., 2019). However, the *Ppara* ^HepKO^ mice on a HFD did have higher plasma triglycerides and ApoB100 levels compared to floxed animals (Stec et al., 2019). The PEG-BR activation of PPARα in obese mice reduced ApoB100 containing VLDL particles in serum, but no effect was observed on *Apob* mRNA expression in the liver. We showed that PPARα in the liver had no impact on *Apob* expression as the hepatic loss in the *Ppara* ^HepKO^ mice on normal chow diet (NFD) or HFD showed no differences (Stec et al., 2019). The hepatic microsomal triglyceride transfer protein (*Mttp*) assists ApoB100 for excretion of the VLDL molecule from the liver (Chen et al., 2008). PEG-BR treated animals had higher *Mttp* levels compared to control. We also found that Mttp levels were low in *Ppara* ^HepKO^ mice, suggesting that Mttp is regulated via PPARα (Stec et al., 2019).

Previous studies have shown a negative correlation between serum total bilirubin levels and blood triglyceride levels (Zhong et al., 2017; Petelin et al., 2020). Interestingly, in obese patients with low serum total bilirubin and higher triglycerides than lean control with bariatric surgery increased plasma bilirubin by two-fold and reduced triglycerides by 50% (Bawahab et al., 2017), illustrating the potential role of bilirubin in triglyceride metabolism. However, these are yet to be tested. Ligands for PPARα effectively reduce plasma triglyceride levels (Srivastava et al., 2006), and plasma ApoB100 containing VLDL (Linden et al., 2002). The bilirubin nanoparticles may be a useful therapy for patients with hypertriglyceridemia. Part of the action of the PEG-BR may be in the reduction of SCD1, which is important for *de novo* production of saturated fatty acids that are contained in ApoB100 VLDL particles (Miyazaki et al., 2000; Flowers and Ntambi, 2009). ApoB100 is a critical component of the VLDL particle that is essential for excretion from the liver (Chen et al., 2008), which carries mostly triglycerides and some cholesterol out of the liver to the blood. The PEG-BR decreased *de novo* lipogenesis and increased fat-burning *β*-oxidation, the latter which provides metabolites for ketone production and secretion of BOHB (Newman and Verdin, 2014b). These findings show a possible role of the bilirubin-PPARα interaction in ketosis, but this has yet to be determined. We have previously used the obesity-induced model with PEG-BR treatments and observed effects on whole-body metabolism (lower plasma glucose and percent fat mass), which could be interrelated to the effects observed in the liver (Gordon et al., 2016).

There is an intriguing accord for BOHB, PPARα, and bilirubin, as they seem to have a metabolic axis that works in concert to control hepatic fat accumulation. The finding that bilirubin may induce the PPARα pathway and increase BHOB production to control hepatic steatosis needs validated by clinical studies. Also, the bilirubin nanoparticles should be used in PPARα knockout mice, preferably tissue-specific KOs, to further validate their mechanisms. Bilirubin is a well-known and highly studied molecule that has been known for centuries as a toxic bile substance, as observed in cases with extremely elevated plasma bilirubin, especially in its unconjugated form, such as that seen with Crigler-Najjar syndrome. Our investigation here posits that bilirubin and the PEG-BR nanoparticle may improve metabolic action and liver function, primarily by reducing liver fat accumulation and NAFLD. We have previously shown that PEG-BR reduced white adipocyte size in obese mice by stimulating PPARα transcriptional activity via regulating its’ coregulator protein interaction (Gordon et al., 2016). Others have shown that the bilirubin nanoparticles protect against hepatic ischemia-reperfusion injury (Kim et al., 2017a), inflammatory lung disease (Kim D. E. et al., 2017), colitis and gut microbiome (Lee et al., 2019), and pancreatic islet xenotransplantation (Kim M. J. et al., 2017). There is promise in bilirubin nanoparticles as a therapeutic, and bilirubin is protective of the cardiovascular system (Hinds and Stec, 2018; Hinds and Stec, 2019) by improving blood pressure (Vera et al., 2009) and renal blood flow (Vera and Stec, 2010). Total bilirubin serum levels were negatively associated with cerebral atherosclerosis, and higher levels had less incidence of extracranial arterial stenosis (ECAS) and intracranial arterial stenosis (ICAS) (Kim et al., 2017b). The protective action of bilirubin may be related to its ability to lower plasma triglycerides, increase fat utilization, promote liver function, and enhance the production of ketones. More studies are required to fully understand the use of bilirubin and bilirubin nanoparticles as a therapeutic for NAFLD and metabolic and cardiovascular disorders.

## Data Availability Statement

The raw data supporting the conclusions of this article will be made available by the authors, without undue reservation.

## Ethics Statement

The animal study was reviewed and approved by Institutional Animal Care and Use Committee of the University of Mississippi Medical Center.

## Author Contributions

TH and DS designed and funded the experiments for the study. JC, DG, DS, and MD performed experiments and obtained the data. TH and DS wrote the manuscript. All authors edited and approved the final version of the manuscript.

## Funding

This work was supported by the National Institutes of Health 1R01DK121797-01A1 (TH) and 1R01DK126884-01 (DS), the National Heart, Lung and Blood Institute K01HL-125445 (TH) and P01 HL05197-11 (DS), and the National Institute of General Medical Sciences P20GM104357-02 (DS). This project was partially supported by Grant Number P30GM122733 (Chemistry and DMPK Core Faculty)-funded by the National Institute of General Medical Sciences (NIGMS) a component of the National Institutes of Health (NIH) as one of its Centers of Biomedical Research Excellence (COBRE). The content is solely the responsibility of the authors and does not necessarily represent the official views of the National Institutes of Health.

## Conflict of Interest

TH and DS have submitted patents on bilirubin and obesity related disorders.

The remaining authors declare that the research was conducted in the absence of any commercial or financial relationships that could be construed as a potential conflict of interest.
