# Supplementary material for: Bilirubin Nanoparticles Reduce Diet-Induced Hepatic Steatosis, Improve Fat Utilization, and Increase Plasma β-Hydroxybutyrate
Source: Front Pharmacol. 2020 Dec 18;11:594574. doi: 10.3389/fphar.2020.594574 (PMC7775678; doi:10.3389/fphar.2020.594574)
Supplement: Supplementary file 1 [file datasheet1.pdf]

Supplemental Figures

Bilirubin nanoparticles reduce hepatic steatosis by fat utilization and production ketone β-hydroxybutyrate

Terry D. Hinds, Jr.[1]*, Justin Creeden[1], Darren M. Gordon[1], Donald F. Stec[2], Matthew C. Donald[3], and David E. Stec[3]*

[1]Department of Pharmacology and Nutritional Sciences, University of Kentucky College of Medicine, Lexington, KY 40508, USA.

[2]Department of Neurosciences, University of Toledo College of Medicine, Toledo, OH 43614 USA.

[3]Small Molecule NMR Facility Core, Vanderbilt Institute of Chemical Biology, Vanderbilt University, Nashville, TN 37235 USA.

[4]Department of Physiology & Biophysics, Cardiorenal and Metabolic Diseases Research Center, University of Mississippi Medical Center, 2500 North State St, Jackson, MS 39216 USA.

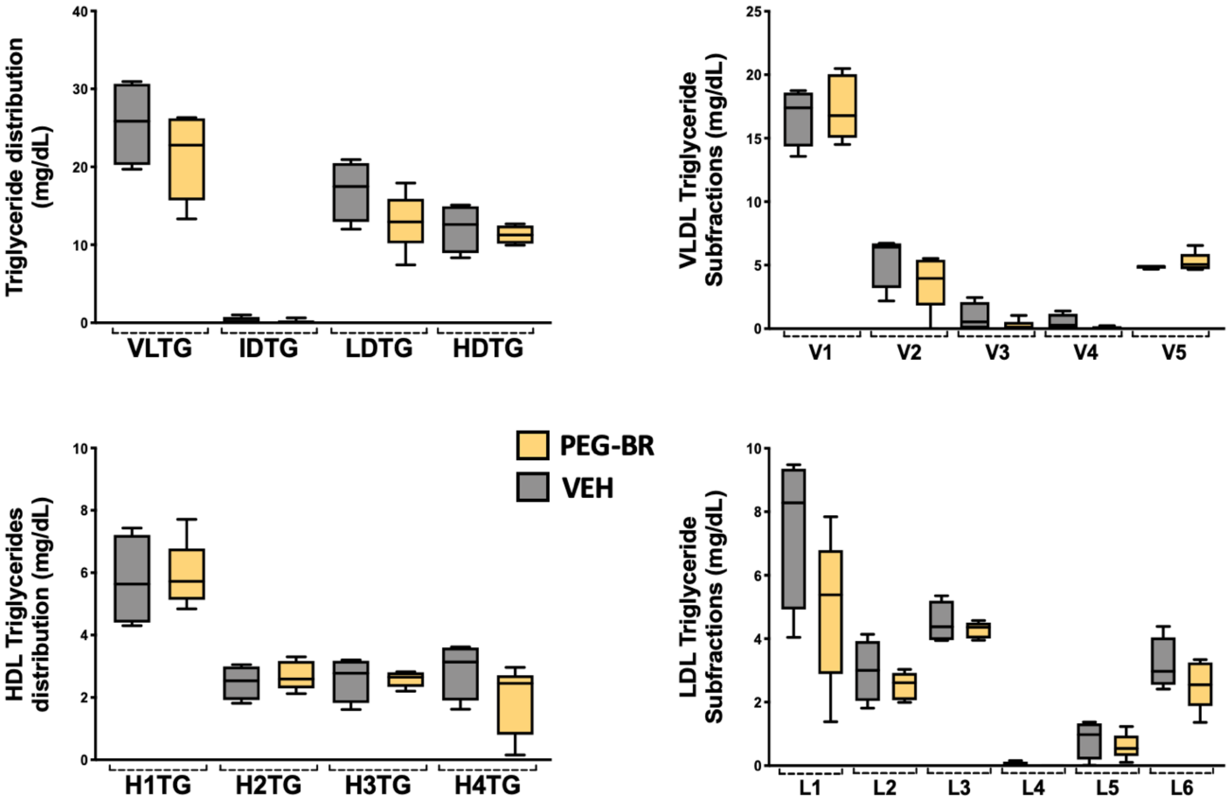

Supplemental Figure 1. Triglyceride distribution and subfractions in the plasma vehicle (VEH) and pegylated bilirubin (PEG-BR) treated mice. Triglyceride distribution (A). High-density lipoprotein (HDL) free triglyceride distribution (B). Very-low density lipoprotein (VLDL) triglyceride distribution (C). Low-density lipoprotein (LDL) triglyceride distribution (D). (VEH, n=4 and PEG-BR, n=5).

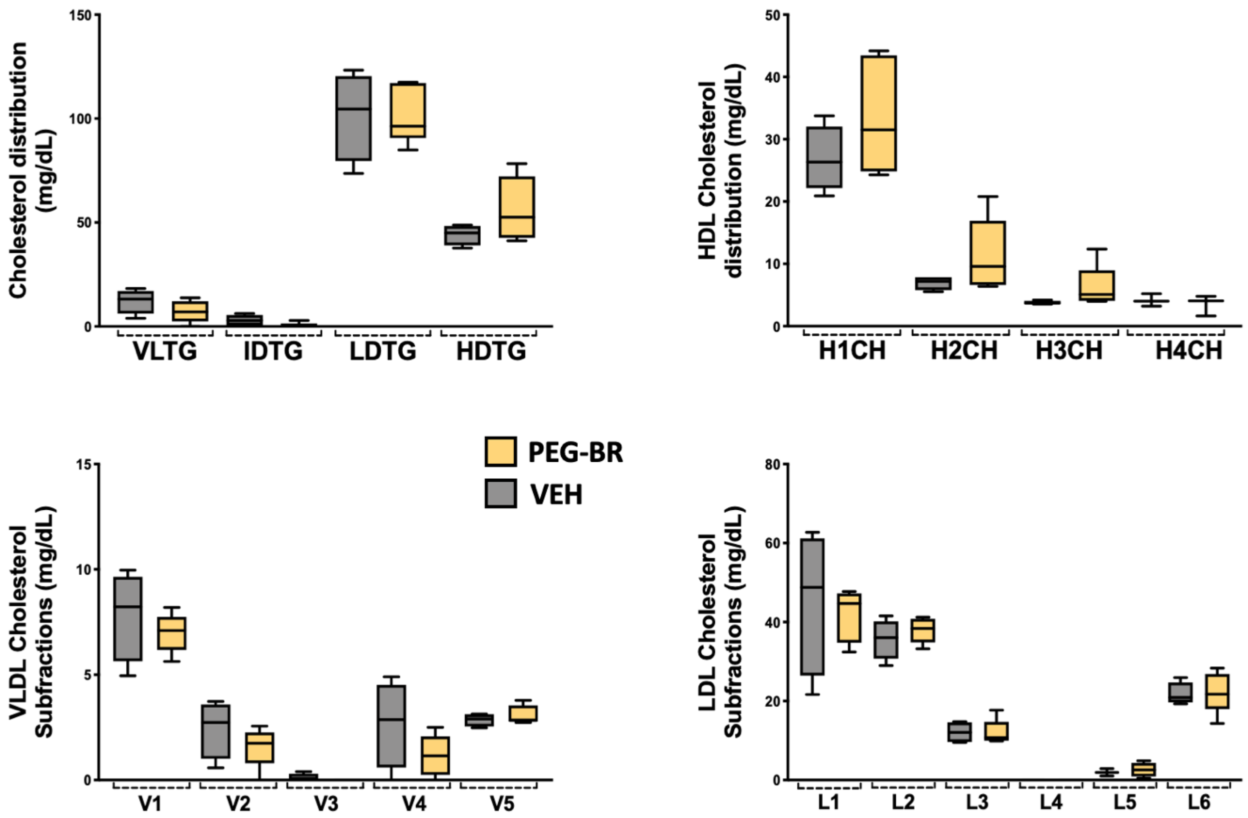

Supplemental Figure 2. Cholesterol distribution and subfractions in the plasma vehicle (VEH) and pegylated bilirubin (PEG-BR) treated mice. Cholesterol distribution (A). High-density lipoprotein (HDL) cholesterol distribution (B). Very-low density lipoprotein (VLDL) cholesterol distribution (C). Low-density lipoprotein (LDL) cholesterol distribution (D). (VEH, n=4 and PEG-BR, n=5).

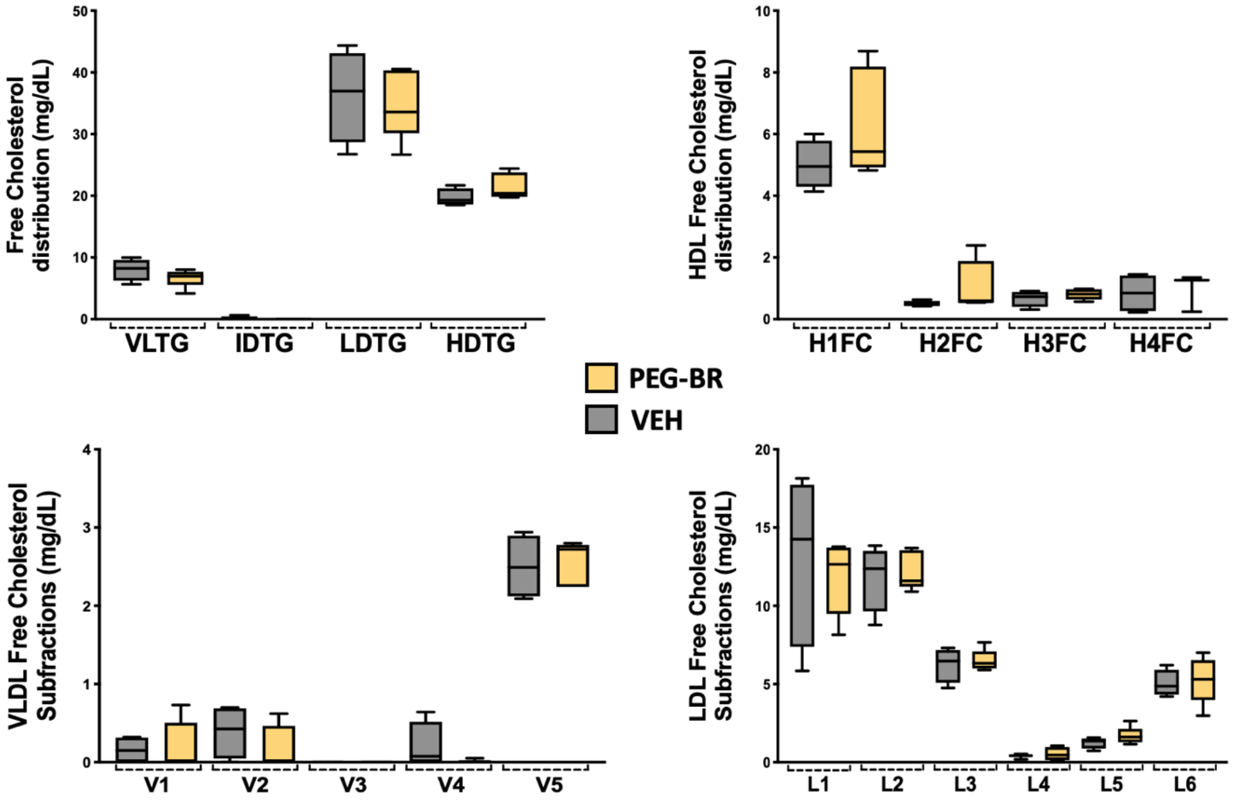

Supplemental Figure 3. Free cholesterol distribution and subfractions in the plasma vehicle (VEH) and pegylated bilirubin (PEG-BR) treated mice. Free Cholesterol distribution (A). High-density lipoprotein (HDL) free cholesterol distribution (B). Very-low density lipoprotein (VLDL) free cholesterol distribution (C). Low-density lipoprotein (LDL) free cholesterol distribution (D). (VEH, n=4 and PEG-BR, n=5).

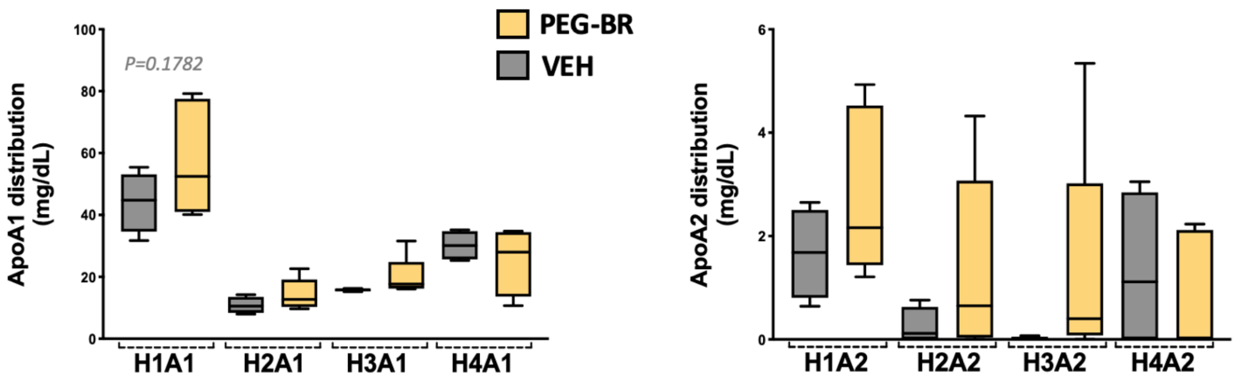

Supplemental Figure 4. Apolipoprotein A (ApoA) subfractions in the plasma vehicle (VEH) and pegylated bilirubin (PEG-BR) treated mice. Apolipoprotein A1 (ApoA1) distribution (A). Apolipoprotein A2 (ApoA2) distribution (B). (VEH, n=4 and PEG-BR, n=5).
